# Neonatal lung-derived SSEA-1^+^ cells exhibited distinct stem/progenitor characteristics and organoid developmental potential

**DOI:** 10.1016/j.isci.2022.104262

**Published:** 2022-04-16

**Authors:** Chien-Chia Liao, Chiao-Juno Chiu, Yao-Hsu Yang, Bor-Luen Chiang

**Affiliations:** 1 Graduate Institute of Immunology, College of Medicine, National Taiwan University, Taipei, Taiwan; 2 Graduate Institute of Clinical Medicine, College of Medicine, National Taiwan University, Taipei, Taiwan; 3 Department of Pediatrics, National Taiwan University Hospital, No. 7 Chung-Shan South Road, Taipei, Taiwan

**Keywords:** Stem cells research, Developmental biology, Tissue Engineering

## Abstract

Stem/progenitor cells, because of their self-renewal and multiple cell type differentiation abilities, have good potential in regenerative medicine. We previously reported a lung epithelial cell population that expressed the stem cell marker SSEA-1 was abundant in neonatal but scarce in adult mice. In the current study, neonatal and adult mouse-derived pulmonary SSEA-1^+^ cells were isolated for further characterization. The results showed that neonatal-derived pulmonary SSEA-1^+^ cells highly expressed lung development-associated genes and had enhanced organoid generation ability compared with the adult cells. Neonatal pulmonary SSEA-1^+^ cells generated airway-like and alveolar-like organoids, suggesting multilineage cell differentiation ability. Organoid generation of neonatal but not adult pulmonary SSEA-1^+^ cells was enhanced by fibroblast growth factor 7 (FGF 7). Furthermore, neonatal pulmonary SSEA-1^+^ cells colonized and developed in decellularized and injured lungs. These results suggest the potential of lung-derived neonatal-stage SSEA-1^+^ cells with enhanced stem/progenitor activity and shed light on future lung engineering applications.

## Highlights

• Pulmonary SSEA-1^+^ cells are abundant in neonatal and scarce in adult stages
• The stem/progenitor activity of pulmonary SSEA-1^+^ cells is enhanced in neonatal stage
• Neonatal pulmonary SSEA-1^+^ cells developed into airway- and alveolar-like organoids
• FGF7 regulates alveolar epithelium development of neonatal pulmonary SSEA-1^+^ cells

## Graphical abstract

**Figure:**
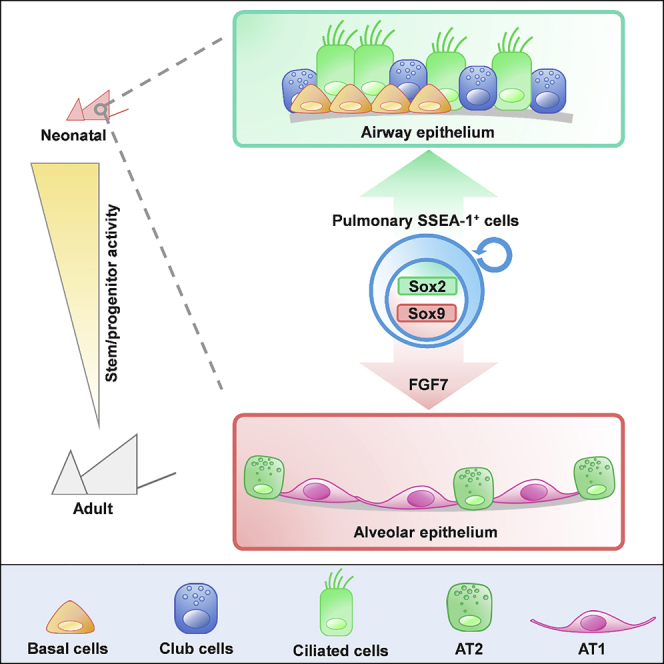

## Introduction

The mature lung epithelium from proximal airways to distal alveoli comprises several cell types that are notably quiescent in the steady state (Kotton and Morrisey, 2014). Many region-specific stem/progenitor cells contribute to epithelial restoration in response to injuries, such as basal cells, club cells, type II alveolar (AT2) cells (Hogan et al., 2014; Whitsett, 2018; Tata and Rajagopal, 2017), bronchioalveolar stem cells (BASCs) (Kim et al., 2005; Schilders et al., 2016), and integrin α6β4^+^ cells (Chapman et al., 2011). Mouse lungs developed from Nkx2.1^+^ endodermal cells at embryonic day 9, which is followed by the pseudoglandular stage, with the airway and alveolar epitheliums developing from Sox2^+^ and Sox9^+^/Id2^+^ progenitors, respectively (Alanis et al., 2014; Yang and Chen, 2014). The lungs continuously grow and separate for alveolar multiplication and maturation up to postnatal days 14–21 (Herriges and Morrisey, 2014). Although studies have revealed that many stem/progenitor cells exist in mature lungs, the differences in stem/progenitor cell properties between developing and mature lungs remain unclear.

Stem/progenitor cells can develop into organoids because of their self-renewal and cell differentiation capacities, and organoids are like miniature versions of the organ of origin (Yin et al., 2016; Barkauskas et al., 2017; Gkatzis et al., 2018; Bar-Ephraim et al., 2020). Studies have revealed that pulmonary stem/progenitor cell-derived organoids, such as basal/club cell-derived tracheal/bronchial organoids (Teisanu et al., 2011; Tadokoro et al., 2014; Danahay et al., 2015; Zheng et al., 2017; Lee et al., 2017), AT2 cell-derived alveolar organoids (Frank et al., 2016; Zepp et al., 2017; Zacharias et al., 2018), and BASC-derived bronchoalveolar organoids (Kim et al., 2005; Schilders et al., 2016), share a similar organization and structure with their origin tissues. Organoid development provides a model for studying stem/progenitor activity, tissue development and repair, and diseases.

Reciprocal communication between epithelium and mesenchyme through growth factor production influences lung development, epithelial cell proliferation and differentiation, and tissue regeneration. Canonical Wnt and fibroblast growth factor (FGFs) signalings are the critical components in driving lung morphogenesis and development (Volckaert and De Langhe, 2015; Danopoulos et al., 2019; Aros et al., 2021). Mice with canonical Wnt signaling inactivation result in impaired proximal-distal patterning and respiratory failure (Mucenski et al., 2003; Shu et al., 2005). FGF10 has been shown to be an essential factor for branch morphogenesis that mice deficiency in *Fgf10* or its receptor *Fgfr2b* results in complete lung agenesis (Sekine et al., 1999; De Moerlooze et al., 2000). In contrast, embryonic lung development was not affected in *Fgf7* knockout mice (Guo et al., 1996). However, FGF7 has been shown to facilitate epithelial cell proliferation and alveolar formation (Padela et al., 2008; Tichelaar et al., 2000). Furthermore, Wnts, FGF7, and FGF10 have also been shown to promote tissue regeneration in response to lung injuries, such as naphthalene-induced airway injury and bleomycin-induced alveolar injury (Yildirim et al., 2008; Gupte et al., 2009; Tanjore et al., 2013; Hsu et al., 2014). Although studies have revealed the roles of growth factors during *in vivo* lung development and tissue regeneration, growth factors that support and regulate the stem/progenitor cell growth and development during *in vitro* culture are not fully understood.

The lungs have constant exposure to airborne environmental factors, and they could be damaged without recovering to their original state in diseases such as chronic obstructive pulmonary disease and idiopathic pulmonary fibrosis. Cell-based therapy is a potential strategy to replace defective cells in regenerative medicine (Kotton and Morrisey, 2014; Parekh et al., 2020). Stem/progenitor cells, because of their self-renewal ability and ability to differentiate into multiple cell types, have excellent potential in regenerative medicine. Stage-specific embryonic antigen-1 (SSEA-1; also known as CD15 and Lewis X) is a carbohydrate-associated molecule that is expressed in murine neutrophils (Kerr and Craig Stocks, 1992; Lucka et al., 2005), embryonic stem cells (Cui et al., 2004; Faherty et al., 2005), and tissue stem/progenitor cells such as neural stem cells (Capela and Temple, 2002; Yanagisawa et al., 2005) and pulmonary stem cells (Ling et al., 2006; Xing et al., 2010). We had previously found a cell population expressing the stem cell marker SSEA-1 in neonatal and adult mouse lungs, the pulmonary SSEA-1^+^ cells. The population of the pulmonary SSEA-1^+^ cells was relatively abundant in neonatal but decreased with age (Chiu et al., 2015). As the lungs are being actively developed during the postnatal period but become quiescent after maturity (Kotton and Morrisey, 2014), this notion raises questions about the activity and properties of stem/progenitor cells derived from neonatal and adult lungs. This study further investigated the difference in characteristics between SSEA-1^+^ pulmonary stem/progenitor cells derived from neonatal and adult lungs. We demonstrated that neonatal pulmonary SSEA-1^+^ cells exhibited increased stem/progenitor properties compared with cells from adults. The results show the potential of neonatal lung-derived SSEA-1^+^ cells in regenerative medicine.

## Results

### Cell surface markers and lineage-associated gene expression in neonatal and adult pulmonary SSEA-1^+^ cells

**Figure 1:**
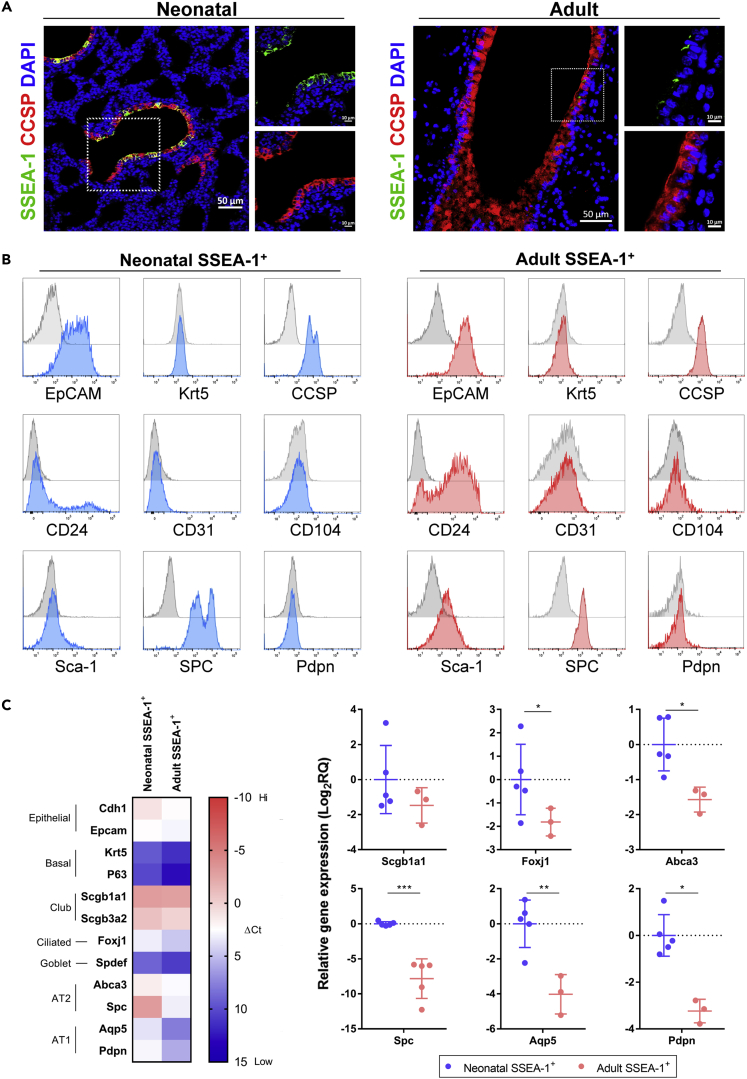
Cell markers and lineage-associated gene expression in neonatal and adult pulmonary SSEA-1^+^ cells (A) Immunofluorescence staining of neonatal (postnatal day 1) and adult (8 weeks old) mice lung sections for stem (SSEA-1) and club (CCSP) cell markers. The nuclei were stained by DAPI. Scale bars are indicated in the figures. (B) FACS analysis of neonatal and adult pulmonary SSEA-1^+^ cells with lung epithelial cell-associated markers. Gray areas, isotype controls. Blue areas, neonatal pulmonary SSEA-1^+^ cells with indicated markers. Red areas, adult pulmonary SSEA-1^+^ cells with indicated markers. (C) Representative qPCR analysis of neonatal and adult pulmonary SSEA-1^+^ cells with lung epithelial cell-associated genes. The ΔCt values were normalized to the *Gapdh* gene and shown by the heatmap in the left panel. The relative gene expression values between neonatal and adult pulmonary SSEA-1^+^ cells are shown in the right panel. Data are represented as mean ± SD from the combination of three to five independent experiments. ∗p < 0.05, ∗∗p < 0.01, ∗∗∗p < 0.001 (Unpaired Student’s *t* test). CCSP, club cell secretory protein. Cdh1, Cadherin-1. EpCAM, epithelial cell adhesion molecule. Krt5, Keratin 5. Pdpn, podoplanin. SPC, surfactant-associated protein C.

In mature lungs, the area from the proximal trachea to the distal alveoli harbors several stem/progenitor cells for local tissue maintenance. However, the properties of these stem/progenitor cells in developing and mature lungs remain unclear. Unlike neonatal lungs, which have active alveolar development, mature lungs are relatively quiescent in the steady state (Kotton and Morrisey, 2014), suggesting that there is an enrichment of the stem/progenitor cell population and its activity in the neonatal stage. To explore potential lung stem/progenitor cells, we previously analyzed the expression of the stem cell marker SSEA-1 in lung epithelial cells, and an SSEA-1-expressing cell population was found to be relatively abundant in neonatal (postnatal day 1) lungs but rare in mature (six to eight weeks of age) lungs (Chiu et al., 2015). In both the neonatal and adult lungs, pulmonary SSEA-1^+^ cells were only observed in the regions rich in club cell marker (club cell secretory protein, CCSP) expression (Figure 1A), revealing the airway localization of the SSEA-1^+^ cells. However, the SSEA-1-expressing cell population and also the SSEA-1 level were decreased in the adult lungs compared to those of neonatal lungs (Figure 1A). For the characterization of SSEA-1^+^ cells, single-cell suspensions from neonatal or adult lung tissues were labeled with anti-SSEA-1 antibodies and enriched by magnetic-based selection. With FACS analysis, both neonatal and adult SSEA-1^+^ cells were found to express epithelial (epithelial cell adhesion molecule, EpCAM), club (CCSP and CD24) (Li et al., 2019), and AT2 (surfactant-associated protein C, SPC) cell markers and lacked expression of basal (keratin 5, Krt5), lung epithelial stem/progenitor cells (CD104) (McQualter et al., 2010; Rabata et al., 2020), AT1 (podoplanin, PDPN), and endothelial (CD31) cell markers (Figure 1B). The coexpression of the club (CCSP) and AT2 (SPC) cell markers by neonatal and adult pulmonary SSEA-1^+^ cells suggested that these cells are not a lineage of the differentiated club or AT2 cells. However, adult pulmonary SSEA-1^+^ cells distinctly expressed Sca-1, a marker of the club and bronchiolar stem/progenitor cells in adult lungs (Zheng et al., 2017; Kim et al., 2005). qPCR analysis revealed that neonatal and adult pulmonary SSEA-1^+^ cells exhibited similar expression patterns of lung epithelial cell-associated genes (Figure 1C). Both neonatal and adult pulmonary SSEA-1^+^ cells highly (ΔCt <0) expressed club cell genes (*Scgb1a1* and *Scgb3a2*) (Figure 1C), but neonatal pulmonary SSEA-1^+^ cells had significantly higher expression levels of ciliated (*Foxj1*), AT2 (*Abca3* and *Spc*), and AT1 (*Aqp5* and *Pdpn*) cell genes than adult pulmonary SSEA-1^+^ cells (Figure 1C). Overall, the neonatal and adult lung-derived SSEA-1^+^ cells shared similar patterns in tissue localization and marker expression, suggesting that neonatal and adult pulmonary SSEA-1^+^ cells are phenotypically similar. So, we further investigated their ability in differentiation and organoid development.

### Neonatal pulmonary SSEA-1^+^ cells exhibited higher stem/progenitor activity than adult cells

**Figure 2:**
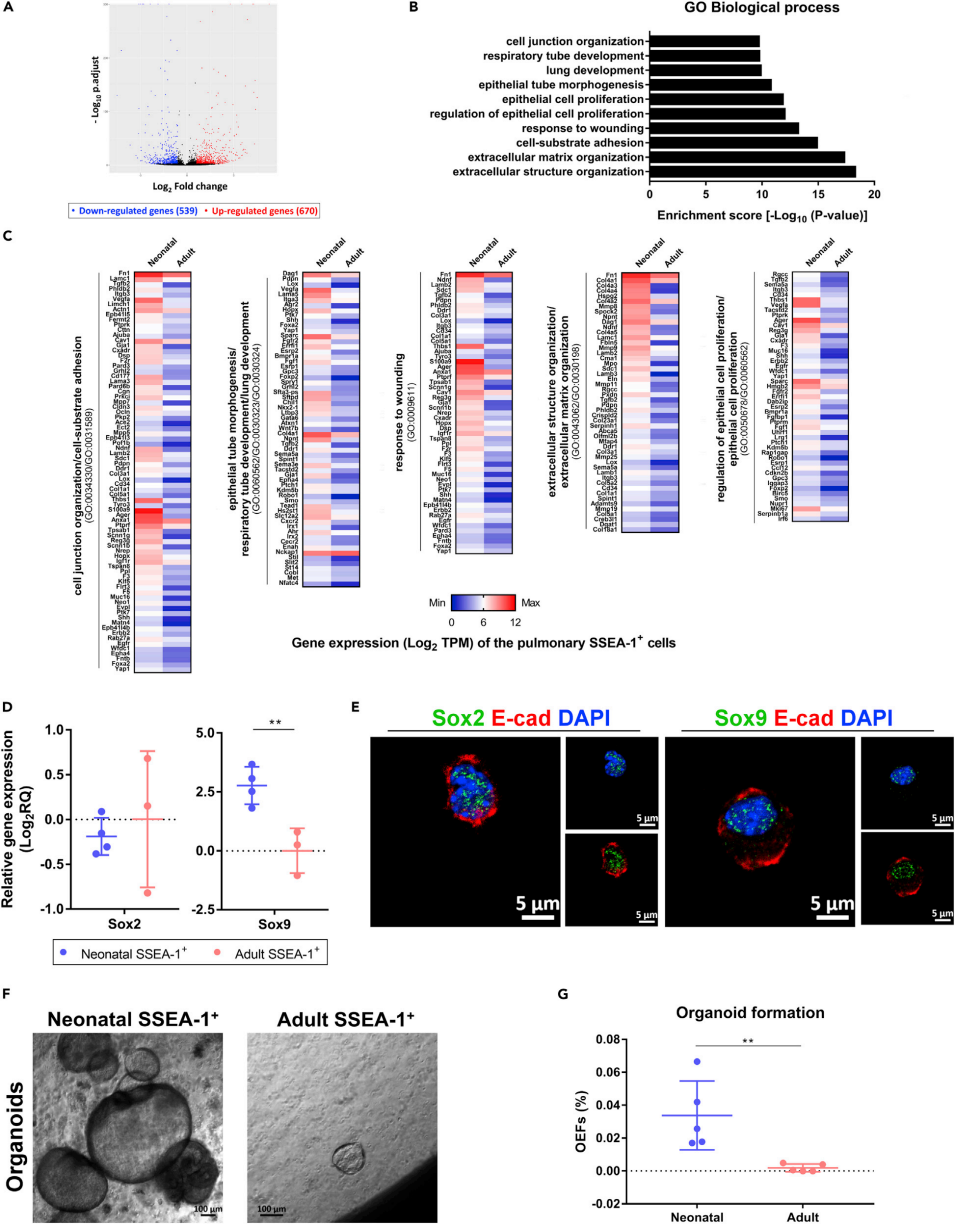
Neonatal pulmonary SSEA-1^+^ cells exhibited higher stem/progenitor activity than adult cells (A–C) Neonatal and adult pulmonary SSEA-1^+^ cells were performed RNA-seq analysis for the transcriptome. The thresholds of DEGs were set by the fold-change ≥2 and p.adjust <0.05. The upregulated DEGs (red dot), downregulated DEGs (blue dot), and normally expressed (black dot) genes of neonatal pulmonary SSEA-1^+^ cells compared to adult pulmonary SSEA-1^+^ cells are shown by volcano plot (A). The enriched terms (p.adjust <0.05) of the biological process from the GO enrichment analysis were based on the DEGs of neonatal pulmonary SSEA-1^+^ cells and shown as enrichment score [-Log_10_ (p value)] (B). The heatmaps of the DEGs from the GO terms listed in (B) are shown by Log_2_ TPM (C). (D) Representative qPCR analysis of neonatal and adult lung-derived SSEA-1^+^ cells with *Sox2* and *Sox9* genes. The ΔCt values were normalized to the *Gapdh* gene. Data are represented as mean ± SD from the combination of three to four independent experiments. ∗∗p < 0.01 (Unpaired Student’s *t* test). (E) Immunofluorescence staining of neonatal pulmonary SSEA-1^+^ cells with indicated markers. The nuclei had stained with DAPI. Scale bar, 5 μm. (F) Neonatal and adult pulmonary SSEA-1^+^cells were embedded in semisolid Matrigel for 3D culture. Images were obtained after 14 days of cell culture. Scale bar, 100 μm. (G) As mentioned in (F), the organoid-forming efficiencies (OEFs) of neonatal and adult pulmonary SSEA-1^+^ cells were calculated as the percentage of the developed organoid number divided by the original seeded cell number. Data are represented as mean ± SD from the combination of five independent experiments. ∗∗p < 0.01 (Unpaired Student’s *t* test). DEGs, differentially expressed genes. E-cad, E-cadherin. GO, gene ontology. TPM, transcript per million. See also Figure S1.

To gain insight into the properties of neonatal and adult lung-derived SSEA-1^+^ cells, RNA sequencing (RNA-seq) was performed to analyze the transcriptome. RNA-seq analysis revealed 1,209 differentially expressed genes (DEGs) (fold-change ≥2 and p.adjust <0.05), of which 670 were upregulated and 539 were downregulated in neonatal pulmonary SSEA-1^+^ cells (Figure 2A). The gene ontology (GO) biological process enrichment analysis revealed that the upregulated DEGs of neonatal pulmonary SSEA-1^+^ cells were enriched in GO terms related to epithelium development (p.adjust <0.05), such as the terms lung development (GO:0030324), epithelial cell proliferation (GO:0050673), and extracellular matrix organization (GO:0030198) (Figures 2B and 2C). These results implied that the developmental potential was increased in neonatal pulmonary SSEA-1^+^ cells. On the other hand, transcription factors play crucial roles in regulating cell behaviors, and Sox2 and Sox9 regulate proximal and distal lung epithelium development, respectively, during the branching morphogenesis of lung development (Akram et al., 2016). The qPCR analysis revealed that the neonatal pulmonary SSEA-1^+^ cells had significantly increased *Sox9* expression compared with the cells from adult mice, and both cell types expressed a comparable level of Sox2 (Figure 2D). FACS analysis also revealed the increased Sox9 expression in neonatal pulmonary SSEA-1^+^ cells (Figure S1). Confocal microscopy verified the expression and nuclear localization of Sox2 and Sox9 in neonatal pulmonary SSEA-1^+^ cells (Figure 2E). Stem/progenitor cells develop into organoids because of their self-renewal and cell differentiation capacities, and the organoids are like miniature versions of the organ of origin (Yin et al., 2016; Lamers et al., 2021; Tindle et al., 2021). To further evaluate the stem/progenitor activity of neonatal and adult pulmonary SSEA-1^+^ cells, cells were embedded in semisolid Matrigel for organoid development. The results showed that neonatal pulmonary SSEA-1^+^ cells generated and developed into organoids during culture (Figures 2F and 2G). In contrast, the adult SSEA-1^+^ cells had significantly reduced organoid generation ability (Figures 2F and 2G). Taken together, these results suggest that the neonatal lung-derived SSEA-1^+^ cells exhibited higher stem/progenitor activities than the cells from adult mice.

### Neonatal lung-derived SSEA-1^+^ cells efficiently developed into airway-like and alveolar-like organoids

**Figure 3:**
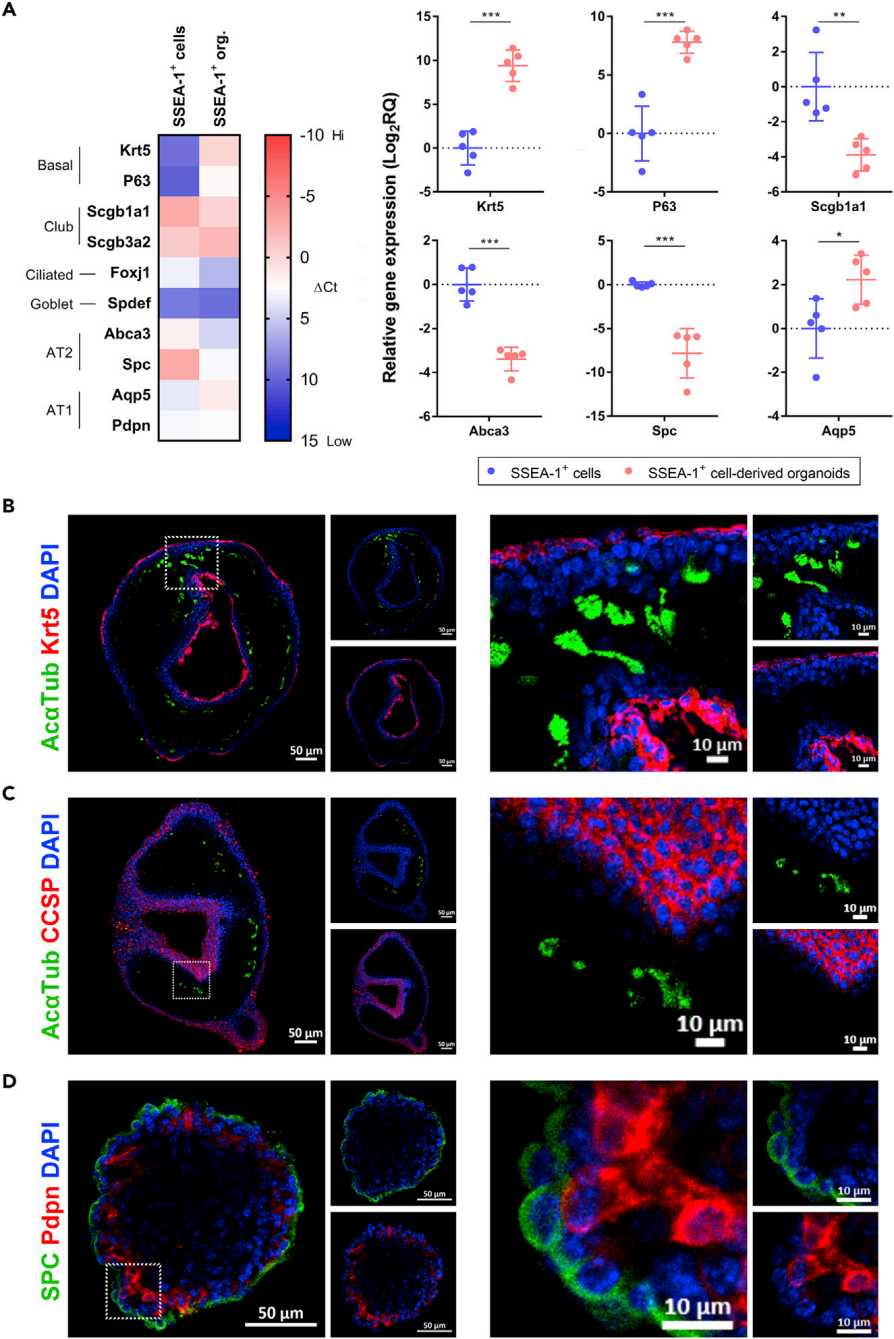
Neonatal pulmonary SSEA-1^+^ cells developed into airway-like and alveolar-like organoids (A) Representative qPCR analysis of neonatal pulmonary SSEA-1^+^ cell-derived organoids with lung epithelial cell-associated genes. The ΔCt values were normalized to the *Gapdh* gene and shown by the heatmap in the left panel. The relative gene expression values between neonatal pulmonary SSEA-1^+^ cells and neonatal pulmonary SSEA-1^+^ cell-derived organoids (SSEA-1^+^ org.) are shown in the right panel. Data are represented as mean ± SD from the combination of five independent experiments. ∗p < 0.05, ∗∗p < 0.01, ∗∗∗p < 0.001 (Unpaired Student’s *t* test). (B–D) Immunofluorescence staining of neonatal pulmonary SSEA-1^+^ cell-derived organoids with indicated markers. The nuclei were stained with DAPI. Scale bars are indicated in the figures. AcαTub, Acetylated α-tubulin. CCSP, club cell secretory protein. Krt5, cytokeratin 5. Pdpn, podoplanin. SPC, surfactant-associated protein C. See also Figure S2.

Organoids are composed of several cell types that constitute structures similar to the organ of origin (Yin et al., 2016). The development of neonatal pulmonary SSEA-1^+^ cell-derived organoids was evaluated by analyzing the expression of lung epithelial-associated genes. The neonatal pulmonary SSEA-1^+^ cell-derived organoids expressed several lung epithelial cell-associated genes that basal (*Krt5* and *P63*), club (*Scgb1a1* and *Scgb3a2*), and AT1 (*Aqp5*) cell genes were highly expressed (ΔCt <0) (Figure 3A). In contrast to the original neonatal pulmonary SSEA-1^+^ cells, neonatal pulmonary SSEA-1^+^ cell-derived organoids had significantly increased basal (*Krt5* and *P63*) and AT1 (*Aqp5*) but decreased club (*Scgb1a1*) and AT2 (*Abca3* and *Spc*) cell gene expression (Figure 3A). These results indicated that there were phenotypic changes in neonatal pulmonary SSEA-1^+^ cells after organoid development. The specific organization of the neonatal pulmonary SSEA-1^+^ cell-derived organoids was analyzed by confocal microscopy. Neonatal pulmonary SSEA-1^+^ cells developed into both airway-like and alveolar-like organoids (Figures 3B–3D). The airway-like organoids were surrounded by basal cells (Krt5^+^ cells) or club cells (CCSP^+^ cells) with the cilia of ciliated cells (AcαTub^+^ cells) distributed in the luminal regions (Figures 3B and 3C). The alveolar-like organoids had exterior AT2 cells (SPC^+^ cells) and interior AT1 cells (Pdpn^+^ cells) (Figure 3D). On the other hand, the developed organoids exhibited specific cellular polarities: the basal, club, and AT2 cells were mostly located in organoid exterior regions, and the ciliated and AT1 cells were distributed in organoid interior areas (Figure S2A). We also observed the alveolar-like organoids exhibited dense morphology with smaller organoid sizes as compared to the airway-like organoids that are constituted with a large luminal structure (Figures 3B–3D and Figure S2C). The morphologies, cellular compositions, and cellular polarities of the neonatal pulmonary SSEA-1^+^ cell-derived airway-like and alveolar-like organoids are similar to the previously described lung organoids, such as the basal- and club cell-derived tracheal/bronchial organoids and the AT2-derived alveolar organoids (Chen et al., 2012; Lee et al., 2017; Danahay et al., 2015). The distinct cellular compositions and interactions could result in the distinct morphologies of the airway-like and alveolar-like organoids. The results suggested the multilineage cell differentiation potential of neonatal pulmonary SSEA-1^+^ cells in the airway and alveolar epithelium development.

### FGF7 regulated neonatal pulmonary SSEA-1^+^ cell activity by increasing organoid generation and AT2 cell development

**Figure 4:**
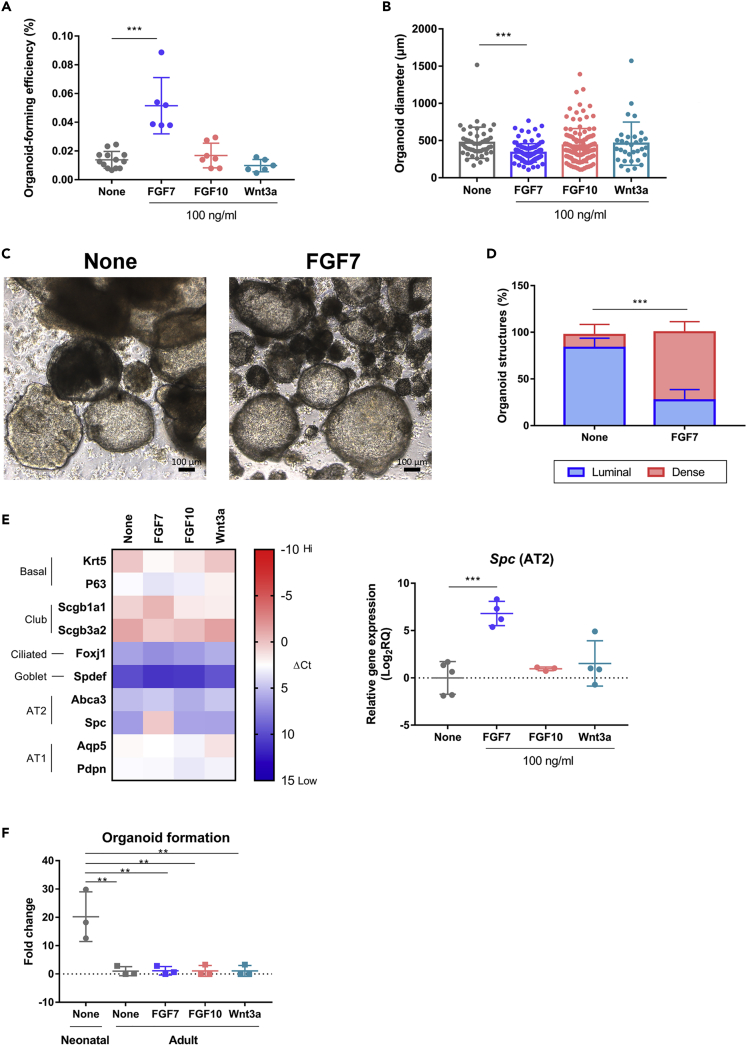
FGF7 regulated the neonatal pulmonary SSEA-1^+^ cell activity by increasing organoid generation and AT2 development (A–E) Neonatal pulmonary SSEA-1^+^ cells were embedded in semisolid Matrigel for 3D culture with the supplementation of FGF7, FGF10, or Wnt3a. The percentage of organoid-forming efficiencies (OEFs) was measured as the developed organoid number divided by the original seeded cell number. Data are represented as mean ± SD from the combination of three to four independent experiments. ∗∗∗p < 0.001 (One-way ANOVA with Dunnett’s test) (A). The diameters of the developed organoids were measured by microscopy with the software ZEN. ∗∗∗p < 0.001 (One-way ANOVA with Dunnett’s test) (B). Images of the organoids developed without any supplementary factors (None) or FGF7 supplementation were obtained after 14 days of cell culture. Scale bar, 100 μm (C). The proportion of the generated luminal and dense morphology organoids between the cells cultured without any supplementary factors (None) or FGF7 supplementation were compared. Data are represented as mean ± SD from the combination of three independent experiments. ∗∗∗p < 0.001 (Two-way ANOVA with Sidak’s test) (D). The developed organoids with lung epithelial-associated gene expression were analyzed by qPCR analysis (ΔCt, normalized to *Gapdh* gene) and shown by the heatmap in the left panel. The relative gene expression values between treatments are shown in the right panel. Data are represented as mean ± SD from the combination of three to five independent experiments. ∗∗∗p < 0.001 (One-way ANOVA with Dunnett’s test) (E).(F) The fold change of neonatal and adult pulmonary SSEA-1^+^ cells in organoid formation between treatments (100 ng/mL) was calculated based on the organoid-forming efficiency of adult pulmonary SSEA-1^+^ cells without any supplementary factors (None). Data are represented as mean ± SD from the combination of three independent experiments. ∗∗p < 0.01 (One-way AVONA with Tukey’s test). See also Figure S3.

The reciprocal signals between the lung epithelium and mesenchyme in the form of growth factors, such as FGFs and Wnts, regulate lung development (Volckaert and De Langhe, 2015). As the neonatal pulmonary SSEA-1^+^ cells spontaneously developed into airway-like or alveolar-like organoids, growth factors that potentially regulate this stem/progenitor cell activity and development were examined. Growth factors, including FGF7, FGF10, and Wnt3a, were supplemented during Matrigel-based 3D culture. The results showed that FGF7 significantly increased the organoid generation activity of neonatal pulmonary SSEA-1^+^ cells (Figure 4A). On the other hand, the organoids developed in the presence of FGF7 had significantly decreased diameters (Figure 4B) that are associated with the increased proportion of small size and dense morphology organoids (Figures 4C and 4D), suggesting the increased alveolar-like organoid development. Expression profiling of lung epithelium-associated genes revealed that there was significantly increased AT2 (*Spc*) cell gene expression in the organoids developed in the presence of FGF7 (Figure 4E). The gene expression profile was not significantly altered in the organoids developed in the presence of FGF10 or Wnt3a supplementation (Figure 4E). And also, the large and luminal structure organoids developed without any supplementary factors were composed of airway epithelial cells such as the basal and ciliated cells (Figure S3A), and the small dense morphology organoids developed under FGF7 supplementation were composed by the alveolar AT2 and AT1 cells (Figure S3B). These results suggested that FGF7 enhanced the organoid generation activity of neonatal pulmonary SSEA-1^+^ cells with the alveolar-like organoid development. Unlike neonatal pulmonary SSEA-1^+^ cells, adult pulmonary SSEA-1^+^ cells did not initiate organoid formation in response to growth factors, including FGF7, FGF10, and Wnt3a (Figure 4F). Taken together, these results suggested a difference in stem/progenitor activity between neonatal and adult lung-derived SSEA-l^+^ cells. Adult pulmonary SSEA-l^+^ cells might become quiescent after the completion of postnatal lung development, and another stimulation is needed to initiate stem/progenitor activity for the development and tissue repair.

### Neonatal pulmonary SSEA-1^+^ cells self-renewed during organoid development

**Figure 5:**
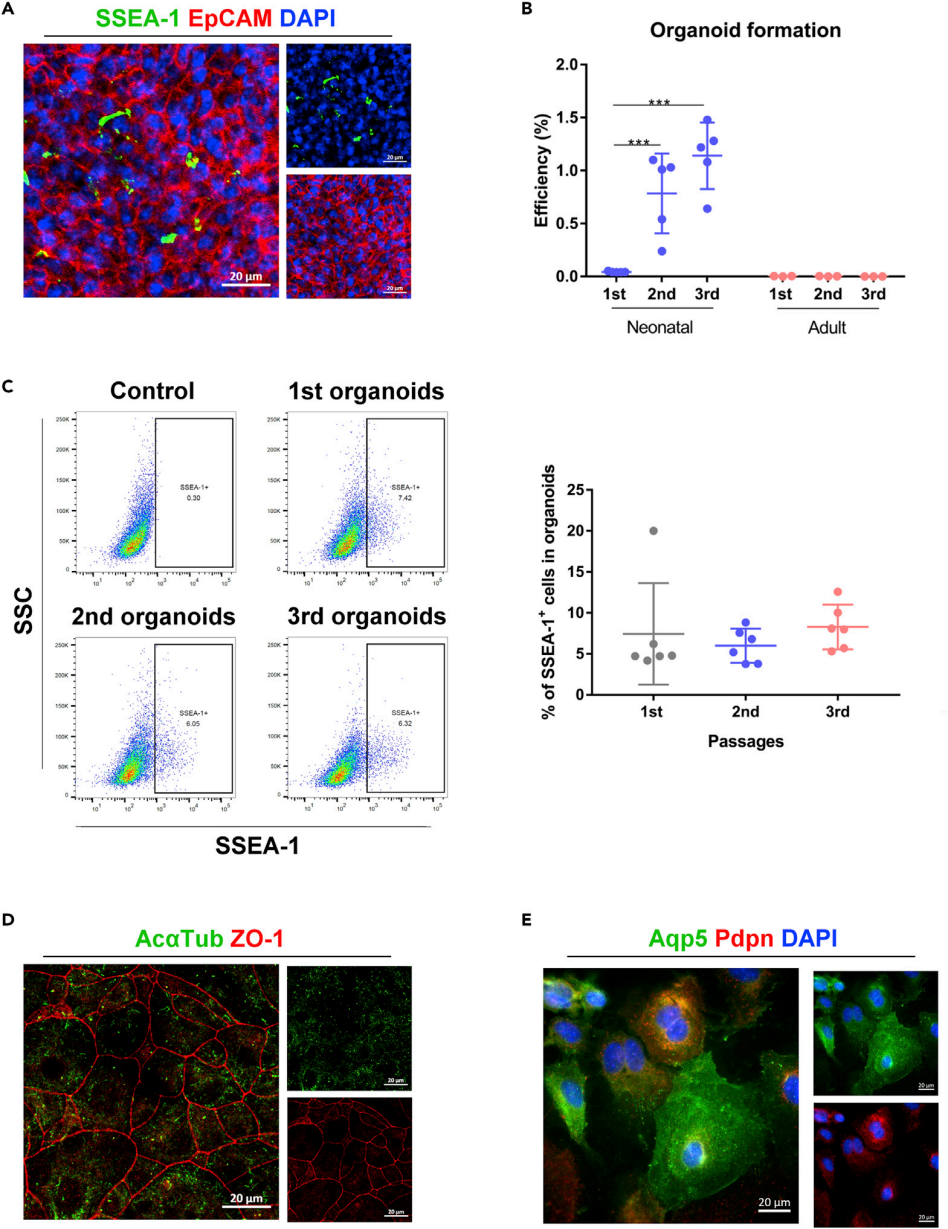
Neonatal pulmonary SSEA-1^+^ cells could be maintained and expanded in the 3D culture system (A) Immunofluorescence staining of neonatal pulmonary SSEA-1^+^ cell-derived organoids with the stem (SSEA-1) and epithelial (EpCAM) cell markers. Scale bar, 20 μm. (B) The organoid-forming efficiencies (OEFs) of neonatal pulmonary SSEA-1^+^ cells between passages were measured as the percentage of the developed organoid number divided by the original seeded cell number. Data are represented as mean ± SD from the combination of three independent experiments. ∗∗∗p < 0.001 (One-way AVONA with Dunnett’s test). (C) The percentage of SSEA-1^+^ cells in neonatal pulmonary SSEA-1^+^ cell-derived organoids between passages was analyzed by FACS with the stem cell marker SSEA-1. Data are represented as mean ± SD from the combination of three to four independent experiments. (One-way AVONA with Tukey’s test). (D and E) Cells from neonatal pulmonary SSEA-1^+^ cell-derived organoids were triggered by trachea epithelial and alveolar cell differentiation. And the differentiated cells were analyzed by immunofluorescence staining with indicated markers. The nuclei were stained with DAPI. Scale bar, 20 μm. AcαTub, Acetylated α-tubulin. Aqp5, aquaporin 5. EpCAM, epithelial cell adhesion molecule. Pdpn, podoplanin. ZO-1, Zona occludens 1. See also Figure S4.

The development of organoids is based on the cell differentiation and self-renewal of stem/progenitor cells. We observed some SSEA-1-expressing cells in the neonatal SSEA-1^+^ cell-derived organoids, as examined by immunofluorescence staining (Figure 5A). The results suggested the maintenance of neonatal pulmonary SSEA-1^+^ cells after organoid development. To further evaluate the self-renewal ability of neonatal pulmonary SSEA-1^+^ cells, the developed organoids were dissociated into single-cell suspensions and passaged with Matrigel-based 3D culture. The results showed that cells from the primary organoids could generate secondary and tertiary organoids with increased organoid generation ability (Figure 5B), and the SSEA-1^+^ cells were also observed at comparable levels in the secondary and tertiary organoids (Figure 5C). Moreover, the SSEA-1^+^ cells isolated from the primary organoids could also generate secondary organoids with increased organoid generation ability (Figures S4A and S4B). These results demonstrated the self-renewal ability of neonatal pulmonary SSEA-1^+^ cells during organoid development. However, the organoid-forming ability of adult pulmonary SSEA-1^+^ cells was not similar to that of neonatal pulmonary SSEA-1^+^ cells during subculture (Figure 5B). On the other hand, cells from neonatal pulmonary SSEA-1^+^ cell-derived organoids triggered the differentiation of tracheal epithelial cells (ZO-1^+^ and AcαTub^+^ cells) (Figure 5D) and alveolar cells (Aqp5^+^ and Pdpn^+^ cells) (Figure 5E). These results suggested that neonatal pulmonary SSEA-1^+^ cells could be expanded in Matrigel-based 3D culture and retained their stem/progenitor properties.

### Therapeutic potential of neonatal pulmonary SSEA-1^+^ cells

**Figure 6:**
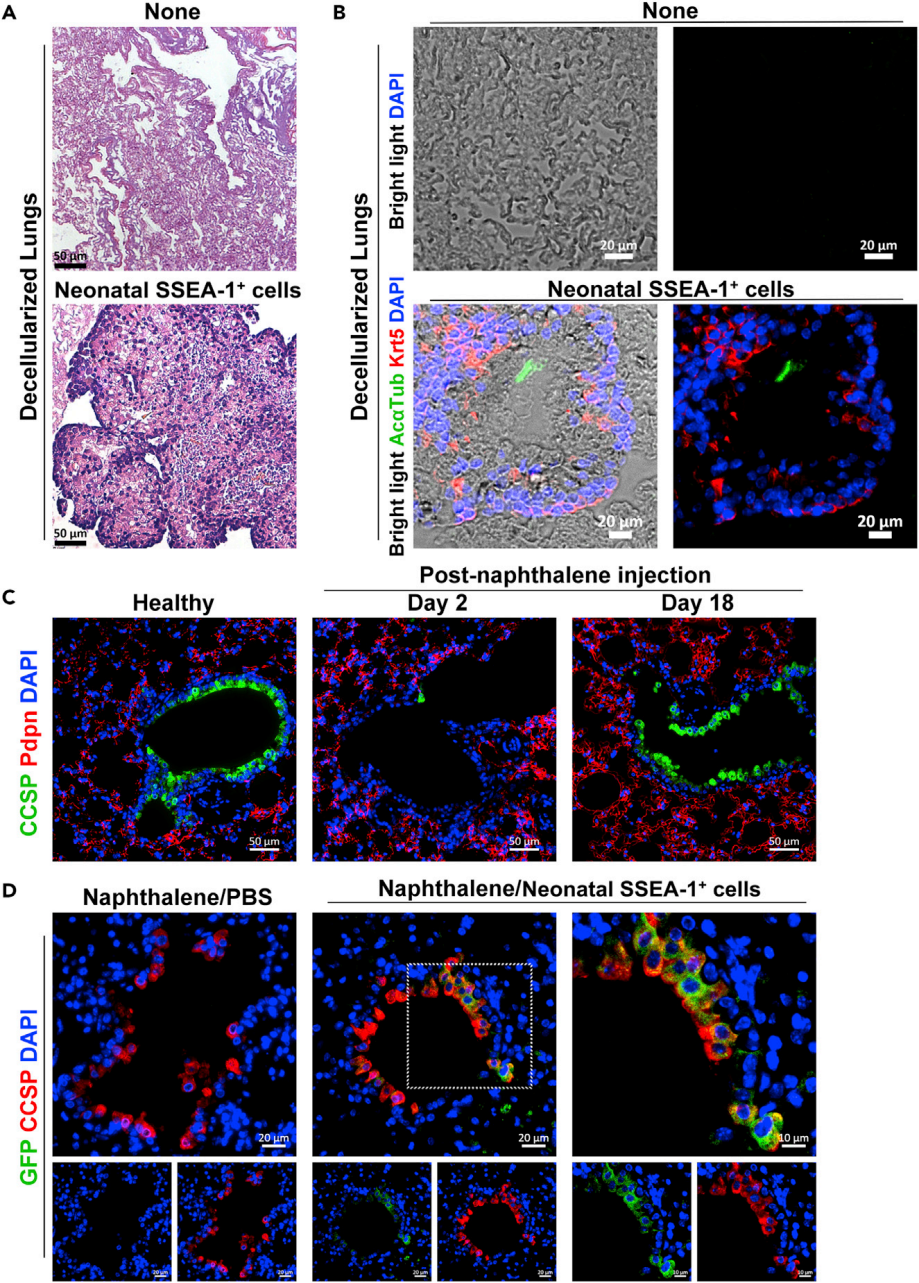
Therapeutic potential of neonatal pulmonary SSEA-1^+^ cells (A and B) Neonatal pulmonary SSEA-1^+^ cells were injected into decellularized lung lobes for incubation. The tissues were analyzed by H&E staining (A) and immunofluorescence staining with indicated markers (B). The nuclei were stained with DAPI. Scale bars are indicated in the figures. Data are represented as one of three independent experiments. (C) Immunofluorescence staining of the lung tissues from healthy and naphthalene-injected mice (post naphthalene injection day 3 and day 18) with the club (CCSP) and AT1 (Pdpn) cell markers. The nuclei were stained with DAPI. Scale bars, 50 μm. (D) Mice with naphthalene injection were intratracheally treated with PBS or GFP-expressing neonatal pulmonary SSEA-1^+^ cells isolated from BALB/c-Tg (PGK1-EGFP) mice. And the lung tissues after 21 days of treatment were analyzed by immunofluorescence staining with club cell marker (CCSP) and GFP. The nuclei were stained with DAPI. Scale bars, 20 μm. Data are represented as one of three independent experiments. AcαTub, Acetylated α-tubulin. CCSP, club cell secretory protein. Krt5, cytokeratin 5. Pdpn, podoplanin. See also Figure S5.

The neonatal pulmonary SSEA-1^+^ cells exhibited stem/progenitor properties, as examined by Matrigel-based 3D culture, and the potential of the cells in tissue regeneration was further evaluated. Acellular lungs were used as scaffolds to analyze the colonization ability of neonatal pulmonary SSEA-1^+^ cells. Adult mouse lungs were perfused with water and detergents to obtain decellularized lungs (Bonenfant et al., 2013), which were injected with neonatal pulmonary SSEA-1^+^ cells and incubated. In comparison to that observed with the acellular lung alone, we found the colonization of neonatal pulmonary SSEA-1^+^ cells on the lung scaffolds (Figure 6A). In addition, some of the lung epithelial cell markers included basal (Krt5), ciliated (AcαTub), club (CCSP), and AT2 (SPC) cells were expressed in the neonatal pulmonary SSEA-1^+^ cell-repopulated lung scaffolds (Figures 6B and S5), suggesting the differentiation of neonatal pulmonary SSEA-1^+^ cells. Next, the naphthalene-induced airway injury mouse model was used to evaluate the potential of neonatal pulmonary SSEA-1^+^ cells in tissue repair. In response to naphthalene injection, we observed that the majority of the club cells but not the alveolar cells were damaged within two days and recovered to a certain extent after eighteen days (Figure 6C). Spontaneous club cell regeneration is aided by resident stem/progenitor cells, such as basal cells (Hong et al., 2004; Hsu et al., 2014) and BASCs (Kim et al., 2005; Liu et al., 2019). Thus, GFP-expressing neonatal pulmonary SSEA-1^+^ cells isolated from BALB/c-Tg (PGK1-EGFP) mice were intratracheally administered on day 2 of naphthalene injection. Three weeks after cell transfer, we observed that some of the regenerated club cells expressed GFP in mice that had received GFP-expressing neonatal pulmonary SSEA-1^+^ cells (Figure 6D). These results suggested that neonatal pulmonary SSEA-1^+^ cells participated in tissue repair in the naphthalene-induced airway injury model. Collectively, the colonization and development of neonatal pulmonary SSEA-1^+^ cells in the acellular and injured lungs suggests the potential of these cells in tissue regeneration.

## Discussion

The mature lungs harbor several region-specific stem/progenitor cells with restricted developmental potential for local tissue maintenance (Hogan et al., 2014; Nadkarni et al., 2016; Tata and Rajagopal, 2017; Barkauskas et al., 2017), and the distal airway epithelium composed of the stem/progenitor cells such as variant club cells and BASCs. Variant club cells, the subpopulation of club cells, distributed around the neuroendocrine bodies and at the bronchoalveolar duct junctions that are characterized by the resistance of the club cell-specific toxin naphthalene due to the lack of cytochrome p4502F2 expression (Giangreco et al., 2002). The BASCs are localized at the bronchoalveolar duct junctions that are characterized by the coexpression of the club (CCSP) and AT2 (SPC) cell markers (Kim et al., 2005). Similar to the features of variant club cells and BASCs, pulmonary SSEA-1^+^ cells were found to be distributed along the airways to the bronchoalveolar duct junctions with the CCSP and SPC coexpression, suggesting the correlation of these cell populations during lung development and tissue regeneration. Studies have suggested the cell surface marker of club cells (EpCAM^+^ CD24^low^ Sca-1^+^) (Li et al., 2019), variant club cells (EpCAM^+^ CD24^low^ Sca-1^-^) (Li et al., 2019), and BASCs (EpCAM^+^ Sca-1^low^ CD24^low^) (Zacharek et al., 2011). We had observed the neonatal and adult pulmonary SSEA-1^+^ cells partially expressed CD24, and the Sca-1 was observed in adults but not in neonatal pulmonary SSEA-1^+^ cells. On the other hand, studies have revealed the heterogeneous of the club (Kathiriya et al., 2020) and BASCs (Liu et al., 2019) by single-cell transcriptome sequencing analysis that suggested the plasticity of the stem/progenitor cells in response to tissue injuries and maintaining the tissue homeostasis. Taken together, the pulmonary SSEA-1^+^ cells shared some but not all the characteristics with the variant club and BASCs.

SSEA-1 is associated with the maintenance of stem cells that the SSEA-1 expression is downregulated upon mouse embryonic stem cell and neural stem cell differentiation (Cui et al., 2004; Faherty et al., 2005; Yagi et al., 2012). Another study revealed that prolonged Sox2 expression reprogrammed the AT2 cells into progenitor-like cells with SPC and CCSP coexpression, and also SSEA-1 expression (Kapere Ochieng et al., 2014). Furthermore, SSEA-1 has been found to be actively involved in the neural stem cell maintenance that knockdown of the SSEA-1 catalyze enzyme Fut9 resulted in the reduced cell proliferation and neurosphere generation (Yagi et al., 2012). These results suggested that SSEA-1 played a role in embryonic and tissue stem/progenitor cell maintenance. Furthermore, administration of Fut9 had increased SSEA-1 expression and cell proliferation resulting in the reverses of hyperoxia-induced cell death, airway inflammation, and lung injury in the murine model of neonatal bronchopulmonary dysplasia, suggesting the roles of SSEA-1 in immunomodulation and tissue regeneration (Chaubey et al., 2021). Studies also suggested that SSEA-1 is an active modulator of Notch signaling for cell proliferation (Yagi et al., 2012; Chaubey et al., 2021). Notch signaling plays a crucial role in pulmonary development and regeneration, including cell fate determination, cell proliferation, and apoptosis (Giuranno et al., 2019). All these studies suggested that SSEA-1 plays a role in regulating stem/progenitor cell maintenance, proliferation, and differentiation. The SSEA-1 expression and the SSEA-1^+^ cell population were found to be relatively abundant in neonatal lungs but scarce in adult lungs (Chiu et al., 2015). Although neonatal and adult pulmonary SSEA-1^+^ cells shared similar lineage gene expression patterns and cell surface markers, neonatal pulmonary SSEA-1^+^ cells exhibited increased activity to develop into organoids. These results suggested that the SSEA-1 moiety might be involved in the stem/progenitor activity of the pulmonary SSEA-1^+^ cells. On the other hand, RNA-seq revealed that GO terms related to epithelial development were enriched in the DEGs of neonatal pulmonary SSEA-1^+^ cells compared to cells from adult mice, including the terms lung development, epithelial proliferation, and extracellular matrix organization. Taken together, the results of the transcriptome and organoid development analyses suggested that neonatal pulmonary SSEA-1^+^ cells exhibited enhanced stem/progenitor activity compared with cells from adult mice.

Studies have revealed the importance of epithelium and mesenchyme interactions through growth factor production that regulates lung development (Volckaert and De Langhe, 2015; Aros et al., 2021). Airway- and alveolar-associated mesenchymal cells exhibited distinct abilities to drive epithelial stem/progenitor cells for airway and alveolar differentiation, respectively (Lee et al., 2017). Studies also indicated the crucial roles of the mesenchymal cells in promoting the stem/progenitor cell self-renewal as revealed by supporting the organoid generation of the club and AT2 cells (Lee et al., 2017; Zepp et al., 2017). Furthermore, alveolar fibroblasts from embryonic, neonatal, and mature lungs have different growth factor levels (Shiraishi et al., 2019), which suggest the pivotal roles of niches or microenvironments in regulating stem/progenitor cell activity. Canonical Wnt and FGFs are the critical growth factors that influence lung development and tissue regeneration (Volckaert and De Langhe, 2015; Aros et al., 2021). FGF7, FGF10, and Wnt3a have been shown to promote lung epithelial stem/progenitor cells in the organoid generation (Frank et al., 2016; Nichane et al., 2017; Zacharias et al., 2018; Rabata et al., 2020). Among the tested growth factors (FGF7, FGF10, and Wnt3a), FGF7 enhanced neonatal pulmonary SSEA-1^+^ cell activity in the organoid generation, but adult SSEA-1^+^ cells had less response to these growth factors. The results were similar to a study that mice with *Fgf7* overexpression showed increased alveolar epithelial cell proliferation and differentiation (Tichelaar et al., 2000). A study has revealed that the signal activation (FGF7 and Notch ligand) and inhibition (BMP4, TGF-β, and GSK-3β) in adult lung-derived AT2 cells are required for fibroblast-free organoid development (Shiraishi et al., 2019). Because the neonatal and adult pulmonary SSEA-1^+^ cells were embedded in Matrigel without any supporting cells for organoid development, these results imply that the stem/progenitor activity of adult lung-derived pulmonary SSEA-1^+^ cells is tightly regulated, like FGF7, FGF10, and Wnt3a are not sufficient to trigger cell activation. On the other hand, inflammatory responses play roles in regulating stem/progenitor behavior and tissue repair (Michael et al., 2016; Wynn and Vannella, 2016; Lloyd and Snelgrove, 2018). In response to influenza virus infection, innate lymphoid cells are critical for restoring airway epithelial cell integrity (Monticelli et al., 2011). During pneumonectomy-induced lung regeneration, the recruitment of myeloid cells with signaling through IL-4Ra is required for compensatory lung growth, suggesting that a regenerative microenvironment with type 2 immunity activates alveolar stem cells for tissue repair in adults (Lechner et al., 2017). In addition, lung tissue-derived macrophages have been shown to play a role to support the organoid generation of adult AT2 cells (Lechner et al., 2017). Further, the macrophage-derived IL-1β primed the AT2 cells into a state that not only facilitates the organoid generation *in vitro* but also promotes alveolar regeneration in response to injury *in vivo* (Choi et al., 2020). All the studies imply that adult pulmonary stem/progenitor cells are quiescent in a steady state and need other stimulation to initiate stem/progenitor activity during tissue injury. The findings also imply the importance of differences in niches and microenvironments between neonatal and adult stages that influence stem/progenitor activity. The precise factors in regulating neonatal and adult pulmonary SSEA-1^+^ cell maintenance, proliferation, and differentiation remain to be elucidated.

Transcription factors act as the central molecules that control cell behaviors and cell fate specification; for example, Sox2 and Sox9/Id2 regulate airway and alveolar epithelium development, respectively (Herriges and Morrisey, 2014). During embryonic lung development, mice with conditional Sox2 mutation exhibit reduced basal, ciliated, and club cell development, indicating the indispensable role of Sox2 in the development of the airway epithelium (Que et al., 2009). In genetic lineage tracing studies of lung pseudoglandular stage development, Sox9^+^ and Id2^+^ progenitors developed not only into alveolar cells but also into Sox2^+^ progenitors (Alanis et al., 2014; Yang and Chen, 2014). Sox9^+^ progenitor cells isolated from E12.5 lungs exhibited multipotency in both airway and alveolar epithelial cell development (Nichane et al., 2017). As Sox2 and Sox9 have crucial roles, the fact that neonatal lung-derived SSEA-1^+^ cells expressed Sox2 and Sox9 and could develop into airway-like and alveolar-like organoids, suggesting their ability to differentiate into multilineage cells. During lung pseudoglandular stage development, human distal epithelial progenitors at the tips have been observed to temporally coexpress Sox2 and Sox9 (Danopoulos et al., 2018). Taken together, these findings suggested that the expression of Sox2 and Sox9 by neonatal lung-derived SSEA-1^+^ cells could be indicative of a transitional state for airway or alveolar epithelium development. Further, Sox9 expression has been observed in the club-like stem/progenitor cells that the cell population was activated and differentiated into alveolar lineages in response to injury (Kathiriya et al., 2020). And Sox9 inactivation affected the proliferative capacity of human lung progenitor cells (Li et al., 2021). Thus, decreased Sox9 expression in adult pulmonary SSEA-1^+^ cells might result in decreased developmental activity or be maintained in a quiescent state.

The normal tissue stem cells including the pulmonary stem cells are maintained in a quiescent state under the homeostatic conditions through the inhibitory signals in cell proliferation and differentiation provided by niches (Hegab et al., 2015; Donne et al., 2015). Studies have revealed that cancer stem cells could be originated from tissue stem cells by the accumulated mutations, leading to self-sufficient and uncontrolled proliferation (Li and Neaves, 2006; Rossi et al., 2020). For example, the bronchioalveolar stem cells could be transformed and give rise to lung adenocarcinoma upon the cells acquiring oncogenic K-ras (G12D) mutation (Kim et al., 2005). Owing to the possibility that the cancer stem cells could be originated from normal tissue stem cells, studies have shown that approximately 73% of current known cancer stem cell markers are also presented by normal embryonic and tissue stem cells, including SSEA-1 (Kim and Ryu, 2017). Because the cell proliferation is tightly regulated in normal tissue stem cells but not in cancer stem cells suggested, the SSEA-1 expression level would be different between these two cell types. Similarly, we had observed the decreased SSEA-1 expression and reduced organoid generation ability of the adult pulmonary SSEA-1^+^ cells that implied the adult pulmonary SSEA-1^+^ cells are normally quiescent under the homeostatic condition. However, it required further studies to identify the expression and the role of SSEA-1 between normal pulmonary stem cells and lung cancer cells.

Tissue-specific stem cells are potential candidates for disease and disorder treatment. However, the properties of lung stem/progenitor cells in developing and mature lungs remain to be clarified. This study investigated pulmonary stem/progenitor cells, SSEA-1^+^ cells, from neonatal and adult mice. The results showed that neonatal pulmonary SSEA-1^+^ cells exhibited stem/progenitor properties related to organoid development and had an enhanced ability compared with adult cells. Furthermore, neonatal pulmonary SSEA-1^+^ cells colonized and repaired decellularized and injured lungs. These findings suggest that there are differences in stem/progenitor cell activities between developing and mature lungs that are important for understanding stem cells as possible therapeutic tools for pulmonary disorder treatment.

### Limitations of the study

Although we showed that the neonatal lung-derived SSEA-1^+^ cells exhibited stem/progenitor activity in both airway and alveolar epithelium development, the study lacks the genetic lineage tracing mouse model to specifically study the role of pulmonary SSEA-1^+^ cells during lung development and tissue regeneration.

## Supporting citations

The following references appear in the supplemental information: Chaboissier et al., 2004; Chung et al., 2013; Holembowski et al., 2014; Horst et al., 2010; Teo et al., 2011; Wang et al., 2013; Yang et al., 2013; Zhang et al., 2016

## STAR★Methods

### Key resources table

**Table:** 

REAGENT or RESOURCE	SOURCE	IDENTIFIER
**Antibodies**		

Anti-SSEA-1 MicroBeads	Miltenyi Biotec	Cat# 130-094-530; RRID: AB_2814656
Anti-SSEA-1 Alexa Fluor 488	BD Biosciences	Cat# 560172; RRID: AB_1645310
Anti-CD324 Alexa Fluor 647	BD Biosciences	Cat# 560062; RRID: AB_1645407
Anti-CD326 APC	BD Biosciences	Cat# 563478; RRID: AB_2738234
Anti-Pdpn PE	BD Biosciences	Cat# 566390; RRID: AB_2739721
Anti-Sox2 Alexa Fluor 647	BD Biosciences	Cat# 560294; RRID: AB_1645324
Anti-acetylated α-tubulin	Sigma-Aldrich	Cat# MABT868; RRID: AB_2819178
Anti-proSPC	Sigma-Aldrich	Cat# AB3786; RRID: AB_91588
Anti-CCSP	Abcam	Cat# ab232562, clone EPR19846
Anti-GFP	Abcam	Cat# ab252881, clone 3H9
Anti-Krt5	Abcam	Cat# ab52635; RRID: AB_869890
Anti-P63	Abcam	Cat# ab735; RRID: AB_305870
Anti-Sox9	Abcam	Cat# ab185230; RRID: AB_2715497
Anti-ZO-1	Abcam	Cat# ab96587; RRID: AB_10680012
Anti-mouse IgG2a Alexa Fluor 488	Invitrogen	Cat # A-21131; AB_2535771
Anti-mouse IgG2b Alexa Fluor 488	Invitrogen	Cat # A-21141; AB_2535778
Anti-rabbit IgG Alexa Fluor 594	Invitrogen	Cat # A-11037; AB_2534095
Anti-rat IgG2a Alexa Fluor 488	Invitrogen	Cat # PA1-84761; AB_933936

**Chemicals, peptides, and recombinant proteins**		

protease-type XIV	Sigma-Aldrich	Cat # P5147
DNase I	Merck	Cat # 11284932001
MEM with EBSS, L-glutamine	Hyclone	Cat # SH30024.02
MCDB201 medium	Sigma-Aldrich	Cat # M6770
Insulin-Transferrin-Selenium	Gibco	Cat # 41400045
Penicillin-streptomycin-amphotericin B	Biological Industries	Cat # 03-033-1B
EGF	Corning	Cat # 354001
FGF7	PeproTech	Cat # 450-60
FGF10	PeproTech	Cat # 450-61
Wnt3a	PeproTech	Cat # 315-20
Growth factor-reduced Matrigel	Corning	Cat # 354230
Y27632 2HCl	Selleckchem	Cat # S1049
Recovery solution	Corning	Cat # 354253
Accumax	STEMCELL	Cat # 07921
Trypsin EDTA	Biological industries	Cat # 03-051-5B
Bovine pituitary extract	Gibco	Cat # 13028041
Bouin’s solution	Sigma-Aldrich	Cat # HT10132
Sodium citrate dihydrate	Sigma-Aldrich	Cat # W302600
Bovine serum albumin	Sigma-Aldrich	Cat # A2153

**Critical commercial assays**		

Transcription factor buffer set	BD Biosciences	Cat # 562725
TRIzol	Invitrogen	Cat# 15596026
MMLV reverse transcriptase	Clontech	Cat # 639524
SYBR™ Green PCR Master Mix	Applied Biosystems™	Cat # 4334973
Naphthalene	Sigma-Aldrich	Cat # 91-20-3
Corn oil	Sigma-Aldrich	Cat # C8267

**Experimental models: Organisms/strains**		

BALB/cByJNarl	National Laboratory Animal Center (NLAC), NARLabs, Taiwan	N/A
BALB/cByJ-Tg (Pgk1-EGFP)01Narl/Narl	National Laboratory Animal Center (NLAC), NARLabs, Taiwan	N/A

**Oligonucleotides**		

Provided in Table S1		

**Software and algorithms**		

Prism 7 software	GraphPad	https://www.graphpad.com
FlowJo software	BD Biosciences	https://www.flowjo.com
ZEN 2.6 (Blue edition)	Zeiss	https://www.zeiss.com

**Other**		

Cell culture inserts, 24-well 0.4 μm pore size	Corning	Cat # 353095

### Resource availability

#### Lead contact

Further information and requests for resources and reagents should be directed to and will be fulfilled by the lead contact, Bor-Luen Chiang (gicmbor@ntu.edu.tw).

#### Materials availability

This study did not generate new unique reagents.

### Experimental model and subject details

#### Animals

BALB/c and BALB/c-Tg (PGK1-EGFP) mice were purchased from the National Laboratory Animal Center (Taiwan) and maintained in the Animal Center of the College of Medicine, National Taiwan University. The animal care and handling protocols have been accepted by the Institutional Animal Care and Use Committee of National Taiwan University. For the naphthalene-induced airway injury mouse model, the female BALB/c mice (8-12 weeks of age) had intraperitoneally injected with naphthalene (Sigma-Aldrich) dissolved in corn oil (Sigma-Aldrich) at a dose of 200 mg/kg body weight. Mice had sacrificed at 3 and 18 days after naphthalene injection. For the cell treatment, mice had intratracheally received neonatal pulmonary SSEA-1^+^ cells (10^6^ cells/mice in 50 μL of PBS) or PBS on day 2 of naphthalene injection, and the mice were sacrificed after 21 days of treatment.

### Method details

#### Preparation of pulmonary SSEA-1^+^ cells

The lung tissues derived from both male and female mice were cut into small pieces and digested by 1 mg/mL of protease-type XIV (Sigma-Aldrich) and 0.5 mg/mL of DNase I (Merck) in MEM (Sigma-Aldrich) for 18 hours at 4°C. PBS contained 5% fetal bovine serum (FBS, Gibco) was added to neutralize the protease activity, and the cells were extracted by repeated pipetting. The cell extract was filtered through a 100-μm nylon mesh (Corning) to obtain single-cell suspensions. The SSEA-1^+^ cells had enriched by anti-SSEA-1 Microbeads (clone MC-480, Miltenyi Biotec) through magnetic separation. The purity of pulmonary SSEA-1^+^ cells was greater than 90% as determined by FACS analysis.

#### Lung epithelial cell differentiation

For the alveolar cell differentiation, cells with 10^5^ cells had seeded on the collagen I-coated 24 well plate. MCDB201 medium (Sigma-Aldrich) supplemented with 5% FBS, Insulin-Transferrin-Selenium (Gibco), 1X penicillin-streptomycin-amphotericin B solution (PSA from 100X stock, Biological Industries), and 25 ng/mL of epidermal growth factor (Corning) were replaced every two days. Cells had differentiated after 7-14 days of culture. For trachea epithelial cell differentiation, cells with 10^5^ cells had seeded on the collagen I-coated 24-well transwell inserts with 0.4 μm pore (Corning). MCDB201 medium supplemented with 5% FBS, Insulin-Transferrin-Selenium, 1X PSA, 0.1 μg/mL of cholera toxin, 30 μg/mL of bovine pituitary extract (Gibco), and 25 ng/mL of epidermal growth factor. Medium in the transwell inserts with 0.4 μm pore and the lower chambers had replaced every two days. As cells reached 100% confluence, cells had maintained under the air-liquid interface by leaving the transwell inserts empty for 10-15 days.

#### Organoid assay

For organoid culture, neonatal and adult pulmonary SSEA-1^+^ cells with 1.5∗10^5^ cells were suspended in 100 μL of 50% growth factor-reduced Matrigel (BD Biosciences) in MCDB201 medium and placed in a 24-well Transwell insert with 0.4 μm pore. Cell-containing Matrigel was allowed to solidify for 15 min at 37°C. MCDB201 medium supplemented with 5% FBS, Insulin-Transferrin-Selenium, 1X PSA, and 25 ng/mL of epidermal growth factor had added in the lower chambers. The Y27632 Rock inhibitor (Selleckchem) had included in the medium during the first two days. The medium had replaced every two days. After 14-21 days of organoid culture, the organoids had collected by the recovery solution (Corning). For self-renewal assay, the organoids were dissociated into single-cell suspensions by Accumax (Innovative Cell Techinologies, Inc) for 2 hours and trypsin (Biological industries) for 2 minutes at room temperature, and 5∗10^4^ cells were embedded in 50% growth factor-reduced Matrigel for culture as mentioned above. The percentage of the organoid-forming efficiency was measured by the organoid number (diameter above 50 μm) from the original seeded cell number.

#### Preparation of decellularized lung scaffolds

The lung decellularization had followed by the protocol (Bonenfant et al., 2013) with some modifications. The heart-lung block had harvested from 6-8 week mice. And the lung decellularization was accompanied by perfusing the lungs with the indicated solution through the trachea and the right ventricle for defined periods. The lungs were perfused three times with 5 mL water solution (deionized water containing 5X PSA) and incubated with water solution for 1 hour at 4°C. After additional three times washing with 5 mL water solution, the lungs were perfused three times with 5 mL Triton solution (0.1% Triton X-100 containing 5X PSA) and incubated with 5 mL Triton solution for 24 hours at 4°C. On day 2, the lungs were removed from Triton solution and washed three times by perfusing with 5 mL water solution. And the lungs were perfused three times with 5 mL SDS solution (2% SDS containing 1X PSA) and incubated with 5 mL SDS solution for 24 hours at 4°C. On day 3, the lungs were removed from the SDS solution and washed three times with 5 mL water solution. The lungs were then perfused and incubated with 5 mL NaCl solution (1 M NaCl containing 5X PSA) for 1 hour at room temperature. After incubation, the lungs had washed three times with 5 mL water solution, and the lungs were then perfused and incubated with 5 mL DNase solution (30 μg/ml DNase, 1.3 mM MgSO_4_, 2 mM CaCl_2_, and 1X PSA in deionized water) for 1 hour at room temperature. Finally, the decellularized lungs were washed three times with 5 mL PBS solution (1X PBS containing 1X PSA) and stored in PBS solution at 4 °C until utilized. For cell culture, neonatal pulmonary SSEA-1^+^ cells with 10^6^ cells were injected into the decellularized lung lobes by 29G syringe (BD Biosciences) and incubated in MCDB201 medium supplemented with 5% FBS, Insulin-Transferrin-Selenium, 1X PSA, and 25 ng/mL of epidermal growth factor for two months.

#### Flow cytometry analysis

For surface molecule staining, cells with 10^5^ cells had stained with the indicated antibodies. For intracellular staining, cells with 10^5^ cells were fixed and permeabilized by a transcription factor buffer set (BD Biosciences) and stained with indicated antibodies. The stained cells had analyzed by FACSLyric^TM^ instrument (BD Biosciences), and data had analyzed by FlowJo software (BD Biosciences).

#### RNA sequencing and data analysis

Total RNA had extracted using TRIzol reagent (Invitrogen) according to the manufacturer’s instructions. RNA Purity and quantification were checked using SimpliNano™ - Biochrom Spectrophotometers (Biochrom, MA, USA). RNA degradation and integrity were monitored by Qsep 100 DNA/RNA Analyzer (BiOptic Inc., Taiwan). Sequencing libraries were generated using KAPA mRNA HyperPrep Kit (KAPA Biosystems, Roche, Basel, Switzerland) with manufacturer’s recommendations, and the index codes were added to attribute sequences to each sample. Sequencing was performed by using an Illumina NovaSeq 6000 platform, and the original data were transformed into raw sequenced reads by CASAVA base calling and stored in FASTQ format. FastQC and MultiQC (Ewels et al., 2016) were used to check fastq files for quality. The obtained raw paired-end reads were filtered by Trimmomatic (v0.38) (Bolger et al., 2014) to discard low-quality reads, trim adaptor sequences, and eliminate poor-quality bases. The obtained high-quality data (clean reads) was used for subsequent analysis. Read pairs from each sample were aligned to the reference genome by the HISAT2 software (v2.1.0) (Kim et al., 2015; Sahraeian et al., 2017). FeatureCounts (v2.0.0) was used to count the reads numbers mapped to individual genes (Liao et al., 2014). For gene expression, the “Trimmed Mean of M-values” normalization (TMM) was performed by DEGseq (v1.40.0) (Wang et al., 2010). Differentially expressed genes (DEGs) analysis was performed in R using DEGseq that was based on the negative binomial distribution (Li et al., 2016; Anders et al., 2013). The resulting p-values were adjusted using Benjamini and Hochberg’s approach for controlling the FDR. GO enrichment analysis of DEGs was conducted using clusterProfiler (v3.14.3) (Yu et al., 2012). GO terms with padj <0.05 were considered significantly enriched.

#### Quantitative polymerase chain reaction

Total RNA had extracted using TRIzol reagent (Invitrogen) according to the manufacturer’s instructions. First-strand cDNA was synthesized with random hexamers by MMLV reverse transcriptase (Clontech). QPCR had perfumed by 7500 FAST Real-Time PCR System (Applied Biosystems) with the SYBR green reagents (Applied Biosystems). The primers used in this study are listed in Table S1. The *Gapdh* had used as an endogenous control.

#### Immunohistochemical staining

Lung tissues had fixed by Bouin’s solution (Sigma-Aldrich) for 24 h, dehydrated in ethanol, and embedded in paraffin wax. Tissue sections with 4 μm thickness were deparaffinized by xylene and rehydrated through graded ethanol. Antigen retrieval had performed by microwave heating in 10 mM sodium citrate (pH 6.0) for 10 min. The non-specific binding of sections had blocked by 3% BSA (Sigma-Aldrich) for 1 h at room temperature. The tissue sections were incubated with indicated primary antibodies overnight at 4°C. After washing, the Alexa Fluor-conjugated secondary antibodies had used to detect the bound primary antibodies for 1 h at room temperature. Confocal microscopy (Zeiss, LSM 510 META, and LSM 880) had performed to visualize the stained cells, and the images had analyzed by ZEN 2.6 (Blue edition) (Zeiss) software.

#### Immunofluorescence staining of the organoids

Cells and organoids were fixed by Bouin’s solution (Sigma-Aldrich) for 10 min at room temperature or fixed by 100% methanol for 10 min at −20°C. The non-specific binding of sections had been blocked by 3% BSA for 1 h at room temperature. Samples were incubated with primary antibodies overnight at 4°C. After washing, the Alexa-Fluor-coupled secondary antibodies (Invitrogen) had used to detect the bound primary antibodies for 1 h at room temperature. Confocal microscopy (ZEISS, LSM 510 META, and LSM780) had performed to visualize the stained cells.

### Quantification and statistical analysis

All statistical analysis had performed by Prism 7.0 (GraphPad Software, San Diego, CA) software. Dual comparisons had made with Student’s *t* test. And experiments with more than two groups had compared by ANOVA with Dunnett’s test or Tukey’s test. Statistical significance is represented by asterisks (∗p ≤ 0.05, ∗∗p ≤ 0.01, ∗∗∗p ≤ 0.001).

## Data Availability

All data reported in this paper will be shared by the lead contact upon request. This paper does not report original code. Any additional information required to reanalyze the data reported in this paper is available from the lead contact upon request.

## Footnotes

Supplemental information can be found online at https://doi.org/10.1016/j.isci.2022.104262.
