# Supplementary material for: Neonatal lung-derived SSEA-1+ cells exhibited distinct stem/progenitor characteristics and organoid developmental potential
Source: iScience. 2022 Apr 16;25(5):104262. doi: 10.1016/j.isci.2022.104262 (PMC9062680; doi:10.1016/j.isci.2022.104262)
Supplement: Document S1. Figures S1–S5 and Table S1 [file mmc1.pdf]

iScience, Volume *25*

Supplemental information

Neonatal lung-derived SSEA-1$^+$ cells exhibited distinct stem/progenitor characteristics and organoid developmental potential

Chien-Chia Liao, Chiao-Juno Chiu, Yao-Hsu Yang, and Bor-Luen Chiang

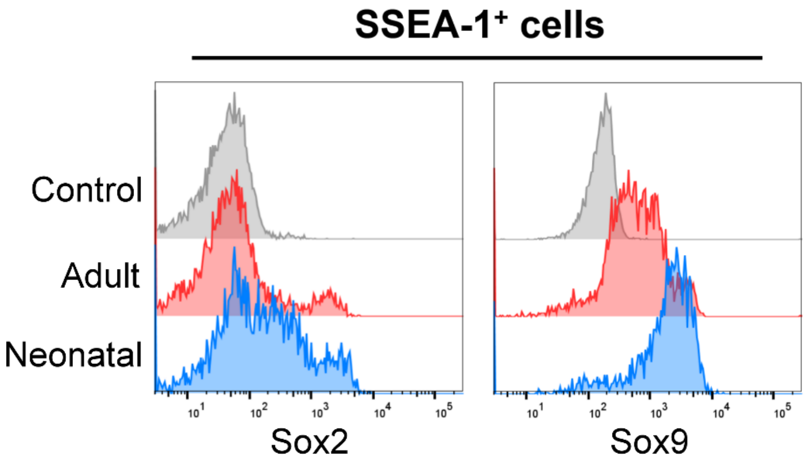

Figure S1. The transcription factor Sox2 and Sox9 expression in neonatal and adult pulmonary SSEA-1^{+} cells, related to Figure 2.

FACS analysis of neonatal and adult pulmonary SSEA-1^{+} cells with transcription factor Sox2 and Sox9. Gray areas, isotype controls. Blue areas, neonatal pulmonary SSEA-1^{+} cells with indicated markers. Red areas, adult pulmonary SSEA-1^{+} cells with indicated markers.

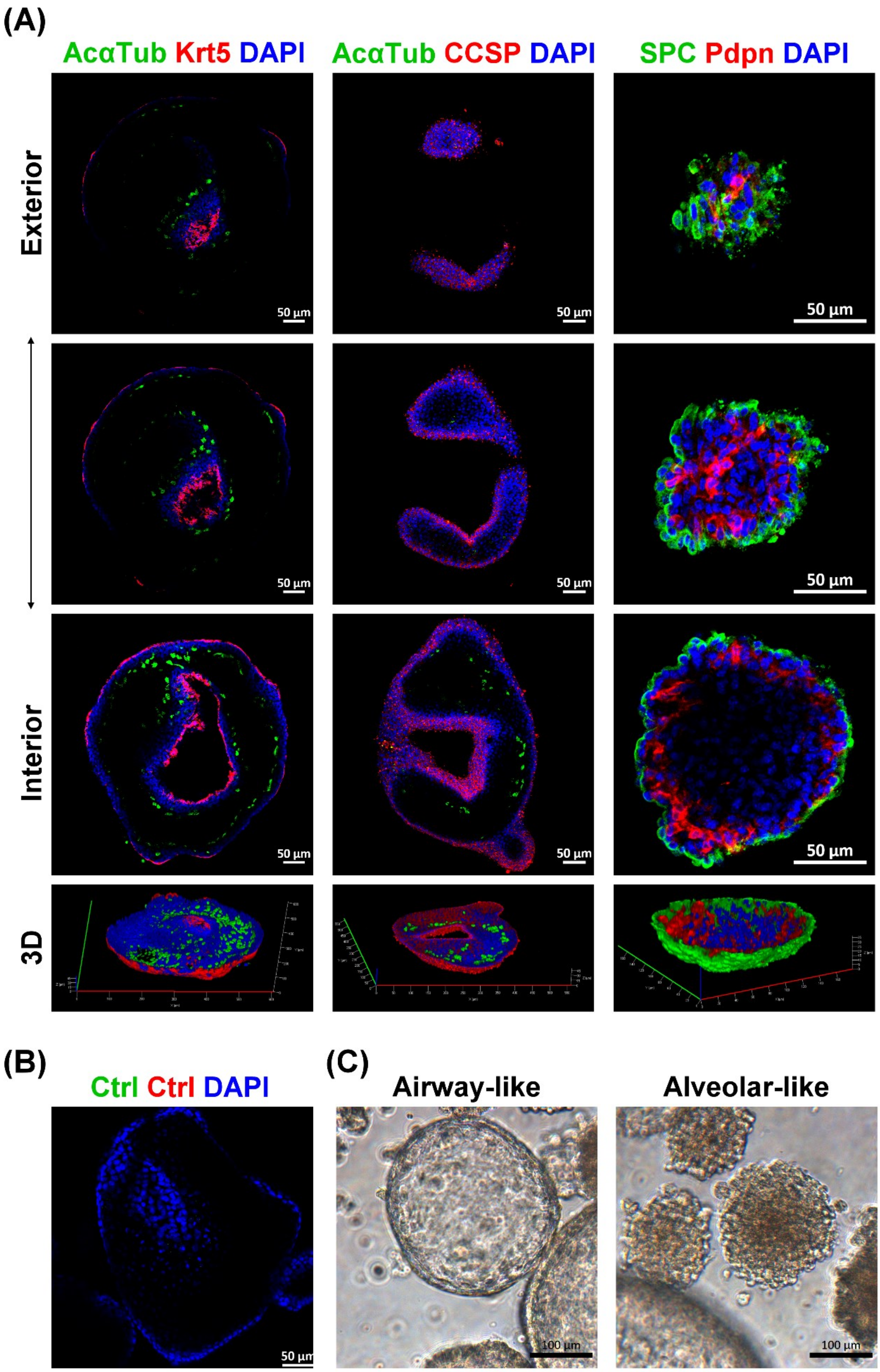

(A)
AcαTub Krt5 DAPI
AcαTub CCSP DAPI
SPC Pdpn DAPI
Exterior
Interior
3D
50 µm
(B)
Ctrl Ctrl DAPI
(C)
Airway-like
Alveolar-like
100 µm

Figure S2. The constitution of neonatal pulmonary SSEA-1$^+$ cell-derived organoids, related to figure 3.

(A and B) Neonatal pulmonary SSEA-1$^+$ cell-derived organoids were immunostained with indicated markers and scanned by confocal microscopy. Sections from exterior to interior regions and the 3D constitutions are shown (A), and the control staining is shown in (B). The nuclei were stained with DAPI. Scale bar, 50 μm.

AcαTub, Acetylated α-tubulin. CCSP, club cell secretory protein. Krt5, Keratin 5. Pdpn, podoplanin. SPC, surfactant associated protein C.

(C) Image of the luminal (airway-like) and dense (alveolar-like) morphology organoids derived from neonatal pulmonary SSEA-1$^+$ cells was obtained after 14 days of cell culture. Scale bar, 100 μm.

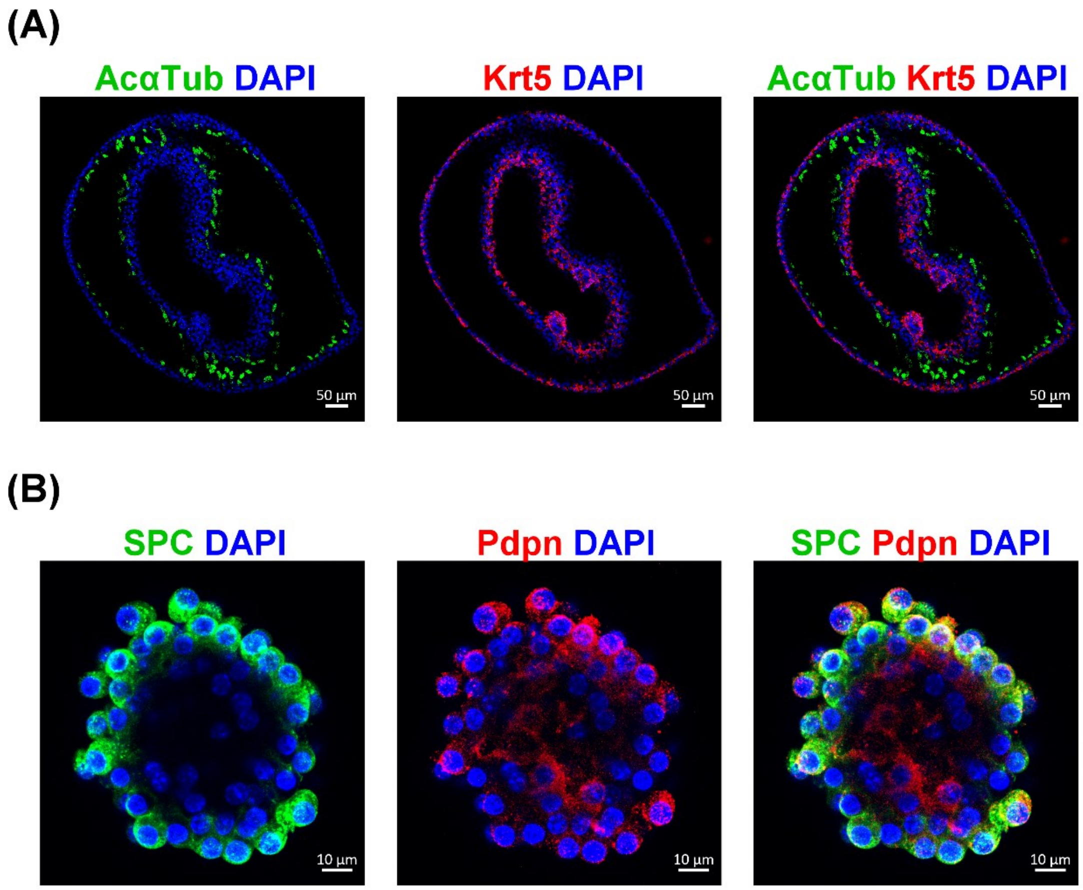

Figure S3. The constitution of neonatal pulmonary SSEA-1$^+$ cell-derived organoids developed with or without FGF7 treatment, related to figure 4.

Immunofluorescence staining of the neonatal pulmonary SSEA-1$^+$ cell-derived organoids developed without any supplementary factors (A) or under FGF7 supplementation (100 ng/ml) (B) with indicated markers. The nuclei were stained with DAPI. Scale bar, indicated in the figures.

AcαTub, Acetylated α-tubulin. Krt5, Keratin 5. Pdpn, podoplanin. SPC, surfactant associated protein C.

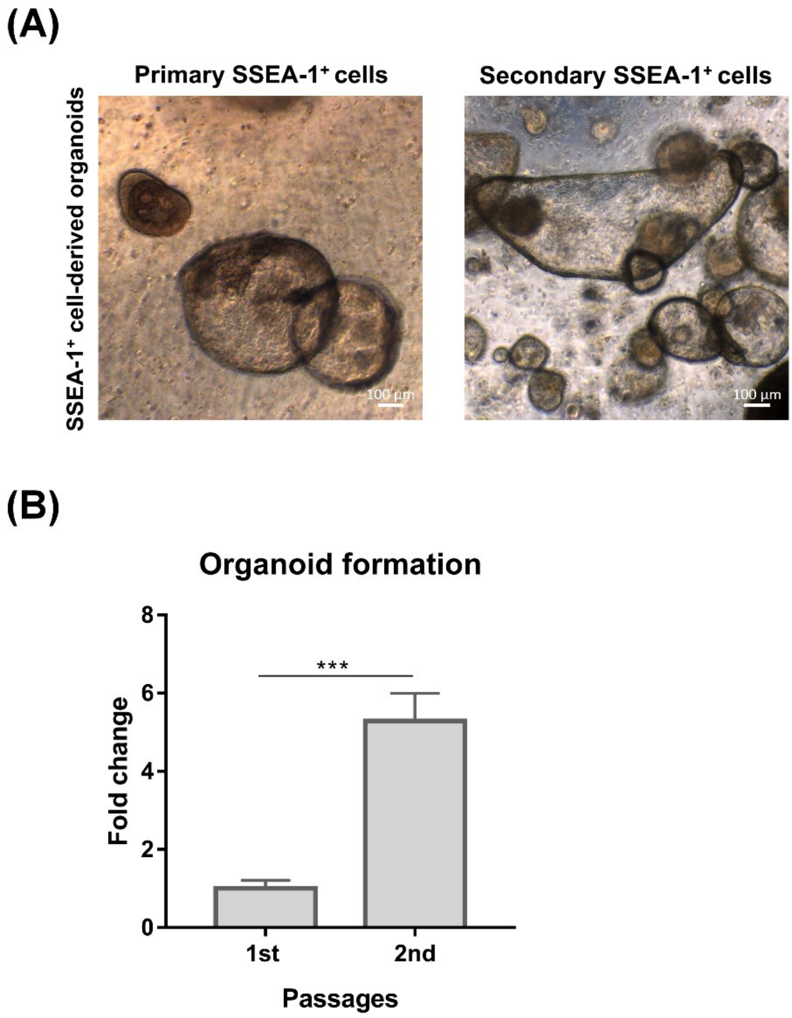

Figure S4. The organoid generation of neonatal pulmonary SSEA-1$^+$ cells during cell passage, related to figure 5.

(A and B) The neonatal pulmonary SSEA-1$^+$ cells were embedded in semisolid Matrigel for organoid development. After 14 days of 3D culture, the primary organoids were dissociated into single-cell suspensions, and the SSEA-1$^+$ cells were isolated for secondary 3D culture. Image of the SSEA-1$^+$ cell-derived primary and secondary organoids was obtained after 14 days of 3D culture. Scale bar, 100 µm (A). The fold change of organoid formation was calculated based on the primary organoid-forming efficiency. Data are represented as mean ± SD. ***$p < 0.001$ (Unpaired Student's t-test) (B).

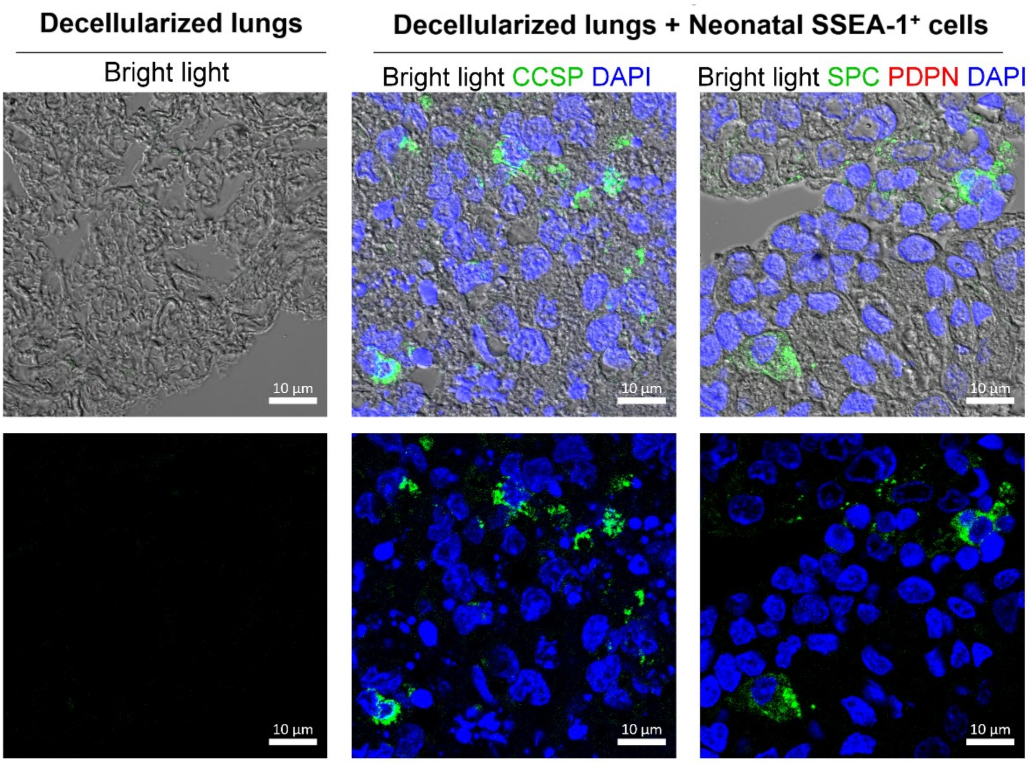

Figure S5. The neonatal pulmonary SSEA-1^{+} cells colonized and developed in the decellularized lungs, related to figure 6.

Neonatal pulmonary SSEA-1^{+} cells were injected into decellularized lung lobes for incubation. The tissues were analyzed by immunofluorescence staining with indicated markers. The nuclei were stained with DAPI. Scale bars, 10 µm.

Table S1. Primers used in this study, related to STAR Methods.

REAGENT or RESOURCE	SOURCE	IDENTIFIER
Epcam forward: aacacaagacgacgtggaca	Zhang et al., 2016	N/A
Epcam: reverse: gctctccgttcactctcagg	Zhang et al., 2016	N/A
Cdh1 forward: actgtgaagggacggtcaac	Nichane et al., 2017	N/A
Cdh1 reverse: ggagcagcaggatcagaatc	Nichane et al., 2017	N/A
Krt5 forward: accttcgaaacaccaagcac	Nichane et al., 2017	N/A
Krt5 reverse: ttggcacactgcttcttgac	Nichane et al., 2017	N/A
P63 forward: cagcaccagcacctacttca	Nichane et al., 2017	N/A
P63 reverse: gataagctggctcacggaag	Nichane et al., 2017	N/A
Scgb1a1 forward: ataccctcccacaagagaccaggata	Wang et al., 2013	N/A
Scgb1a1 reverse: acacagggcagtgacaaggcttta	Wang et al., 2013	N/A
Scgb3a2 forward: gctggtatctatctttctgctggtg	Wang et al., 2013	N/A
Scgb3a2 reverse: acaacagggagacggttgatgaga	Wang et al., 2013	N/A
Foxj1 forward: acacgtgaagccaccctact	This paper	N/A
Foxj1 reverse: tgttcaaggacaggttgtgg	This paper	N/A
Spdef forward: ttggatgagcactcgctaga	Horst et al., 2010	N/A
Spdef reverse: agccggtactggtgttctgt	Horst et al., 2010	N/A
Abca3 forward: cagctcaccctcctactctg	Chung et al., 2013	N/A
Abca3 reverse: actggatcttcaagcgaagcc	Chung et al., 2013	N/A
Spc forward: atgagtagcaaagaggtcctg	Yang et al., 2013	N/A
Spc reverse: tggtgtctgctcgctcactc	Yang et al., 2013	N/A
Aqp5 forward: tctcctccacggactcccgc	This paper	N/A
Aqp5 reverse: ggcccccacgatcggtccta	This paper	N/A
Pdpn forward: gaggaactgtccacctcagc	Holembowski et al., 2014	N/A
Pdpn reverse: tggctaacaagacgccaact	Holembowski et al., 2014	N/A
Sox2 forward: agatgcacaactcggagatcag	Teo et al., 2011	N/A
Sox2 reverse: tcatgagcgtcttggttttcc	Teo et al., 2011	N/A
Sox9 forward: gacaagcggaggccgaa	Chaboissier et al., 2004	N/A
Sox9 reverse: ccagcttgcacgtcggtt	Chaboissier et al., 2004	N/A
Gapdh forward: gatgggtgtgaaccacg	This paper	N/A
Gapdh reverse: agatccacgacggacac	This paper	N/A
